# Network-based social capital and capacity-building programs: an example from Ethiopia

**DOI:** 10.1186/1478-4491-8-17

**Published:** 2010-07-01

**Authors:** Shoba Ramanadhan, Sosena Kebede, Jeannie Mantopoulos, Elizabeth H Bradley

**Affiliations:** 1 Center for Community-Based Research, Dana-Farber Cancer Institute, 44 Binney St., LW 703, Boston, MA 02115 USA; 2 Division of Health Policy and Administration, Yale School of Public Health, 60 College St. P.O. Box 208034, New Haven, CT 06520 USA

## Abstract

**Introduction:**

Capacity-building programs are vital for healthcare workforce development in low- and middle-income countries. In addition to increasing human capital, participation in such programs may lead to new professional networks and access to social capital. Although network development and social capital generation were not explicit program goals, we took advantage of a natural experiment and studied the social networks that developed in the first year of an executive-education Master of Hospital and Healthcare Administration (MHA) program in Jimma, Ethiopia.

**Case description:**

We conducted a sociometric network analysis, which included all program participants and supporters (formally affiliated educators and mentors). We studied two networks: the Trainee Network (all 25 trainees) and the Trainee-Supporter Network (25 trainees and 38 supporters). The independent variable of interest was out-degree, the number of program-related connections reported by each respondent. We assessed social capital exchange in terms of resource exchange, both informational and functional. Contingency table analysis for relational data was used to evaluate the relationship between out-degree and informational and functional exchange.

**Discussion and evaluation:**

Both networks demonstrated growth and inclusion of most or all network members. In the Trainee Network, those with the highest level of out-degree had the highest reports of informational exchange, χ^2 ^(1, *N *= 23) = 123.61, p < 0.01. We did not find a statistically significant relationship between out-degree and functional exchange in this network, χ^2^(1, *N *= 23) = 26.11, p > 0.05. In the Trainee-Supporter Network, trainees with the highest level of out-degree had the highest reports of informational exchange, χ^2 ^(1, *N *= 23) = 74.93, p < 0.05. The same pattern held for functional exchange, χ^2 ^(1, *N *= 23) = 81.31, p < 0.01.

**Conclusions:**

We found substantial and productive development of social networks in the first year of a healthcare management capacity-building program. Environmental constraints, such as limited access to information and communication technologies, or challenges with transportation and logistics, may limit the ability of some participants to engage in the networks fully. This work suggests that intentional social network development may be an important opportunity for capacity-building programs as healthcare systems improve their ability to manage resources and tackle emerging problems.

## Introduction

The global health agenda is increasingly focused on strengthening health systems to improve population-level health outcomes in low- and middle-income countries [1]. One component of this strategy focuses on the development of sufficient workforce capacity, a target area that has been somewhat resistant to intervention thus far [2,3]. The chronic shortage of skilled leadership in the healthcare sectors of low- and middle-income countries greatly hinders the improvement of facilities and systems and the ability to provide needed services [2,4-6].

Successful management and leadership training programs have improved process-related outcomes (such as planning and coordination, delivery of services, and resource management) in a range of countries, including The Gambia, Ethiopia, and Nicaragua [7-9]. Such capacity-building programs typically target human capital, or increased value of a professional from acquiring knowledge, skills, and other assets that may benefit an employer or system. Another benefit of these programs, which is seldom evaluated, may be the development of social capital, or resources that exist in a social structure and can be retrieved and utilized to meet specific goals [10].

Taking a broad view of potential benefits is consistent with current perspectives on capacity-building, which focus on processes that assist individuals, organizations, and societies in efforts to manage, develop, and utilize the resources at their disposal to solve problems [3,11], here those related to healthcare. This view represents an intentional shift away from programs focused on technical assistance and knowledge transfer towards an endogenous process, owned and driven by those who will ultimately benefit from and sustain changes in their systems [3]. Capacity-building program participants (and the organizations for which they work) can benefit from increased social capital as participants are able to utilize relationships to increase their effectiveness and performance [10,12,13]. In this way, participants can leverage relationships to improve communication and collaboration across and within organizations to reach a common goal [14,15]. Such benefits are particularly important in low-resource settings as organizations are expected to turn to external sources to find needed resources [16].

### A network perspective on social capital

Although there are a wide range of conceptualizations of social capital [17], we take a network perspective, which holds that the extent to which an individual can realize the benefits of social capital is a function of that individual's position in a given social network [10,18]. This drives our focus on: a) the resources that can be accessed by network members (either directly or through contacts), and b) the structure of relationships or linkages in a network of interest [10]. In a professional network, key benefits of increased social capital among colleagues include increased exchange of information and resources [17,19]. For example, sharing of appropriate and timely information allows individuals to make strategic adjustments to reach their goals [10,20]. Additionally, participants can access novel information by developing relationships with individuals who are dissimilar in terms of experience and professional contacts [21]. By learning in the context of social relationships, network members can come together to identify pressing problems, make sense of complex changes in the environment, and develop innovative solutions [22,23]. Provision of tangible support or material resources from one network member to another also improves network members' performance [24]. By tapping into relationships, network members can gain access to contacts' resources, and perhaps more importantly, to the resources held by the organization(s) represented by those contacts [25]. The challenge is to balance efficiency (knowing others who have contacts and resources that are very different than one's own) and effectiveness (development of a strong set of key contacts) [18].

Social network analysis provides the necessary tools for our analysis as the methodology allows for the assessment of structures in social relationships, as well as the resources exchanged through those relationships [26]. Additionally, given that successful capacity-building relies on changes at the individual, organizational, and system levels [27], the ability to assess relationships and resource flow at multiple levels allows for a holistic assessment. For example, a network in which all members are connected prompts members to develop trust and a sense of obligation towards each other and encourages the generation of social capital [28]. At the same time, at the individual level, connections to other network members are expected to provide new access to resources for program participants. If a capacity-building program results in network structures that support resource exchange, network-based social capital can have an impact on the ultimate goal of management training programs: the improvement of trainee performance.

Despite the number of programs focused on building healthcare worker capacity [2,7-9] and the understanding that increased collaboration and partnerships are important outcomes of capacity-building efforts [29], we are not aware of previous studies examining how such programs may affect the structure and functioning of resulting social networks. Examining this potential impact is important to our understanding of the full impact of capacity-building programs in health. Using survey data from hospital executives participating in an executive-education program in Ethiopia [30], we conducted a social network analysis to examine the growth of the network and the social capital generated by the network (in the form of resource exchange) during the first year of the program. Social network development and social capital generation were not explicit goals of the training program, but we were able to take advantage of this natural experiment to test exploratory hypotheses. We expected to find growth and resource exchange within networks as well as a positive association between network connections and resource exchange. We tested these assumptions among a network of program participants and among a network of participants plus educators and mentors participating in the program.

## Case description

### Study setting

The capacity-building program under study was a two-year executive-education Master of Hospital and Healthcare Administration (MHA) program in Ethiopia developed by the Federal Ministry of Health (FMOH), the Clinton HIV/AIDS Initiative (CHAI), Jimma University, and the Yale School of Public Health [9,31]. The program was implemented at the request of the FMOH, with the goal of developing skilled executives to improve hospital management in Ethiopia, a low-resource, high-demand setting. This program was part of a larger quality improvement effort targeting the Ethiopian healthcare system, which began decentralization in 1994. The course was offered by Jimma University in Jimma, Ethiopia and was the first graduate-level program for hospital management in the country. The course was administered and taught jointly by faculty from Jimma and Yale Universities, with local coordination provided by a Program Director and Program Assistant. As an executive-education program, the course was offered over two years, with three-week long sessions in residence three times per year, as well as regular progress reports and evaluations when trainees were working at their hospitals.

Executives of public hospitals were eligible to apply. The course focused on improving trainees' skills in a range of management-related areas, such as human resources, hospital operations, financial management, strategic planning, and leadership. Trainees also had the opportunity to develop professional connections with each other as well as with leaders and mentors in Ethiopia and the United States.

### Study design and respondents

We conducted a cross-sectional study at the end of the first year of the MHA program to describe the social networks that developed during the year. Data were collected with a self-administered survey of two groups of respondents: trainees and supporters. Trainees were the first Chief Executive Officers (CEOs) of public hospitals in Ethiopia. Supporters comprised educators and mentors formally linked with the MHA program through either Yale or Jimma University or through CHAI. We contacted all 25 trainees enrolled in the MHA program and 38 supporters affiliated with the program to complete the survey. All research procedures were approved by the Human Investigation Committee at the Yale School of Public Health and the Institutional Review Board at Jimma University.

### Data collection and measures

The self-administered survey was distributed in December 2008 and January 2009 and required approximately 20 minutes to complete. Paper copies of the survey were distributed to all trainees in residence during the December course session and electronic copies were distributed to all other respondents. Surveys were administered in English, which was the language of instruction and a requirement for participants in the MHA program.

For this study, we focused on two networks: 1) the Trainee Network, which was comprised solely of trainees, and 2) the Trainee-Supporter Network, which included trainees and supporters (educators and mentors). Respondents were presented with a roster that listed all trainees and supporters. The survey asked all respondents to identify trainees and supporters with whom they interacted for professional purposes. Respondents also noted whether or not they were acquainted with each network member before the MHA program started. From these responses, we derived our measures of interest for each network.

We measured a series of network characteristics which have been shown in other settings to promote exchange of information and flow through networks [26]. These measures were based on data about connections (or reported relationships) between network members. Some measures focus on presence or absence of a connection, whereas others include information about the 'direction' of the connection. For the latter, the measure can capture whether Member X reported a connection to Member Y, Y reported a connection to X, or both reported a connection to each other.

To describe the network as a whole, the first measure of interest was network density, or the proportion of possible relationships between members that were realized, which described the extent to which network members are connected, regardless of the direction of connections [26]. A more dense, or more highly connected, network may be useful for sharing information and resources and cooperation, whereas a more sparsely connected network may provide greater access to diverse contacts and novel resources [10,18]. A density level of around 15-20% is expected to support knowledge-sharing in a network of about 100 members [32]. We also identified isolates, individuals who reported no connections to other network members. Isolates are of interest as their lack of connections prevents them from contributing to or benefiting from network membership. Last, we identified components, or subgroups of members that are not connected to each other and therefore cannot share information and resources between subgroups [26].

Shifting our focus to individual network members, we calculated degree, the number of connections between a given network member and all other network members, regardless of the direction of ties [33]. The bulk of our analyses focused on out-degree, or connections from a given network member to other network members. Thus, if Member X reported three connections with other network members, that member's out-degree value would be three, regardless of how many network members reported connections to Member X. Compared with degree, this measure narrows the focus to connections that may be perceived as functionally useful to respondents [34]; here, these connections involve the set of individuals from whom respondents may seek and gain skills. In the Trainee Network, 'trainee out-degree' was the number of connections a trainee reported regarding other trainees, grouped into tertiles. In the Trainee-Supporter Network, 'trainee-supporter out-degree' was the number of connections to supporters reported by each trainee, grouped into tertiles. Last, geographic homophily referred to whether or not pairs of network members worked in the same region.

To assess potential by-products of social network development, we measured informational and functional exchanges, which are complementary manifestations of social capital that can help trainees achieve work-related goals [10,24]. Informational exchange refers to access to necessary knowledge, the ability to transmit it to the correct person, and acquisition of information with sufficient time to react [18]. Trainees were asked whether or not they received guidance in non-classroom settings from: a) other trainees, and b) supporters on a series of subjects. These topics included: problem-solving, human resources, finance management and budgeting, basic public health, biostatistics/research methods, hospital operations, strategic management, health policy development and analysis, health ethics and public health law, leadership, and management information or tools. The list of topics was defined in the curriculum as critical to the program and most topics, but not all, were covered in the MHA course at the time of the survey. We created a summary score of the total number of exchanges reported and dichotomized responses at the 50^th ^percentile for each network, resulting in categories of 'low exchange' and 'high exchange' for each network. Based on the distribution of data, 'low exchange represents zero reported informational exchange in the Trainee Network.

Functional exchange described the provision of tangible support from one network member to another [24]. Such exchange often involves collaboration between institutions or individuals that benefit one party to a greater degree, e.g., one individual training another on the use of a new tool. Examples of tangible support can include sharing of useful tools, policies, and materials or serving as a reference for colleagues [25,35]. Trainees were asked whether or not they received a series of tangible resources from: a) other trainees, and b) supporters. These resources included: materials and goods (such as surplus supplies), connections/introductions, and hands-on instruction, such as through site visits. We created a summary score of the total number of exchanges reported and dichotomized responses at the 50^th ^percentile for each network, resulting in categories of 'low exchange' and 'high exchange' for each network.

### Analysis

We conducted a sociometric network analysis for both the 25-member trainee network and for the larger 63-member trainee-supporter network, which included educators and mentors (n = 38) in addition to trainees (n = 25). Sociometric analyses assess the connections between all members of each network of interest, supporting evaluation of network growth and resource exchange [36,37]. Thus, an individual who was invited to participate, but did not fill out a survey, could have been noted as a contact by another respondent and would still appear in the dataset. Although the Trainee Network is wholly contained within the Trainee-Supporter Network, we analyzed them separately to be able to isolate resource exchange among complementary sets of ties that are important for trainees.

Network analysis requires dedicated software to assess relational data, and we used UCINET-6 [38]. As network data are not independent and do not meet the assumptions of classical statistical techniques, we utilized procedures developed for network data available in the UCINET software package [38,39]. Thus, the significance tests were based on random permutations of matrices as is appropriate for relational data. Here, the significance levels were determined based on distributions created from 10 000 random permutations. The analytic procedures also supported comparison of matrices of data. Descriptive measures were calculated using standard UCINET procedures developed for network data. We utilized UCINET Contingency Table Analysis to assess the association of out-degree with two types of resource exchange. We tested the relationship between geographic homophily and connection patterns using UCINET QAP Relational Cross-Tabulation.

## Results

### Trainee network

Among trainees, 23 of 25 individuals completed the survey (92% response rate). Table 1 describes the characteristics of trainees' hospitals. The trainee hospitals had on average 204 beds with a range of 40-800 beds, and the average number of employees per hospital was 399 employees, with a range of 82-2500 employees. The majority of hospitals (72%) were classified as regional; one-third were rural.

**Table 1:** Descriptive characteristics for hospitals led by trainees (n = 25).

	n	Range
**Hospital location**		

Rural	8 (32%)	

Urban	17 (68%)	

Number of beds: mean	204	40-800

Number of employees: mean	399	82-2500

Hospital classification		

Federal	4 (16%)	

Regional	18 (72%)	

Sub-regional/Zonal	3 (12%)	

**Figure 1:**
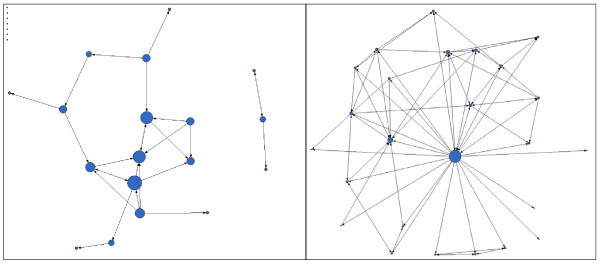
**MHA trainee network before the program started (left) and at year 1 (right), n = 25**. Key: Circular nodes represent trainees. Node size represents degree (number of connections); nodes in upper left corner of diagram on left represent isolates (individuals who did not report any connections).

The network graphs comparing connections before the program started at year 1 (Figure 1) and key network measures (Table 2) demonstrate network-level growth. The network transitioned from having seven isolates (individuals who were not connected to anyone) and two components (distinct and isolated subgroups) to having zero isolates and only one component. At year 1, the network demonstrated closure, or the ability of all members to connect with each other, either directly or through contacts. The density of connections increased from 4% to 13% of all potential connections over the year. In terms of resource exchange, 55% of trainees reported that they had informational exchanges with other trainees during the first year of the program. The same percentage reported functional exchange with other trainees. We found that trainee out-degree (the number of connections reported by the trainee regarding other trainees) increased from 1.0 to 3.0 connections in the first year of the program, which was not a statistically significant increase. We found increased variation in trainee out-degree and trainee in-degree values at year 1 compared with the beginning of the program, suggesting that the network became more centralized, or more centred on a subset of individuals.

**Table 2:** Descriptive measures for the trainee-only sociometric network (25-member network)

Measure	Pre-MHA	Year 1
*Network-level measures*		

Density (proportion of potential ties that were actually realized)	0.04	0.13

Isolates (members of the network not connected to anyone else)	7	0

Components (distinct and isolated subgroups in the network)	2	0

*Individual-level measures*		

Degree (all connections reported to/from the respondent)	Mean: 1.92	Mean: 4.88
	SD: 1.79	SD: 4.42

Trainee out-degree (number of connections reported by respondent regarding others)	Mean: 1.04	Mean:3.00
	SD: 1.43	SD: 4.62

Trainee in-degree (number of connections reported regarding respondent by others)	Mean: 1.04	Mean: 3.00
	SD: 1.25	SD: 1.67


At year 1, trainees in the lowest out-degree tertile averaged 0.5 outgoing connections compared with an average of 2.0 outgoing connections for the middle tertile, and 6.1 outgoing connections for the highest tertile. Individuals with the highest level of connections were more likely to be working in the capital city of Addis Ababa compared with other regions (Fisher's exact test, p = 0.03). We found a significant (p < 0.001) association between regional homophily and connections reported at year 1. Of potential connections among individuals from the same region, 45% (45 of 100) were reported compared with 6% (30 of 500) of potential connections among individuals from different regions.

As presented in Table 3, we found that at year 1, trainee out-degree was positively associated with informational exchange, χ^2^(1, *N *= 23) = 123.61, p < 0.01. Those with the highest tertile of trainee out-degree had the highest reports of informational exchange. We did not find a statistically significant relationship between trainee out-degree and functional exchange, χ^2^(1, *N *= 23) = 26.11, p > 0.05.

**Table 3:** Relationship between trainee out-degree and resource exchange at year 1, contingency table analysis (n = 23).

	Informational: no exchange (%)	Informational: some exchange (%)	Functional: no exchange (%)	Functional: some exchange (%)
Trainee out-degree				

Low	70.00	8.33	40.00	33.33

Medium	10.00	33.33	30.00	16.67

High	20.00	58.33	30.00	50.00

				

Observed X^2^	123.61**		26.11	

Key: ~ < 0.10, * p < 0.05, ** p < 0.01, *** p < 0.001

### Trainee-Supporter Network

**Figure 2:**
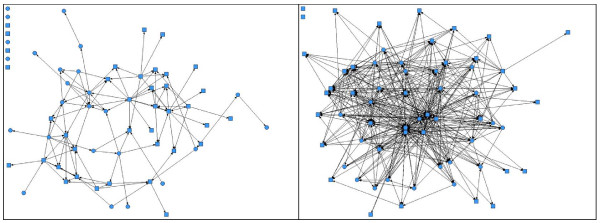
**Trainee-supporter network before the program started (left) and at year 1 (right), n = 63**. Key: Square nodes represent supporters, circular nodes represent trainees. Nodes in upper left corner of diagrams represent isolates (individuals who did not report any connections).

For the larger network, 41 of 63 individuals completed the survey (65% response rate), with a 47% response rate among supporters. Network-level growth was assessed using a pair of network graphs (Figure 2) and a series of complementary measures (Table 4). The density increased from 3% to 13% of all potential ties realized over the first year of the program. We analyzed density increases among subgroups and found increased ties from trainees to supporters (3% to 20%), from supporters to trainees (0% to 12%) and from supporters to supporters (5% to 9%). The number of isolates decreased from 8 to 2 in this network, and there was only one component at year 1, ignoring isolates. Again, increased variation in out-degree and in-degree values for the full network from the beginning of the program to year 1 suggests that the network became more centralized. Assessing the overall network, the individuals with the most connections in this network were mainly faculty and staff that played a central role in program administration and teaching.

**Table 4:** Descriptive measures for the trainee-supporter sociometric network (63-member network).

Measure	Pre-MHA	Year 1
*Network-level measures*		

Density (proportion of potential ties that were actually realized)	0.03	0.13

Density between and within groups of trainees and supporters	Ties among trainees: 0.04	Ties among trainees: 0.13
	Ties from trainees to supporters: 0.03	Ties from trainees to supporters: 0.20
	Ties from supporters to trainees: 0.00	Ties from supporters to trainees: 0.12
	Ties among supporters: 0.05	Ties among supporters: 0.09

Isolates (members of the network not connected to anyone else)	8 isolates	2 isolates

Components (distinct and isolated subgroups in the network)	1 component + isolates	1 component + isolates

*Individual-level measures*		

Degree (all connections reported to/from the respondent)	Mean: 3.52	Mean: 14.22
	SD: 3.11	SD: 10.87

Out-degree (number of connections reported by respondent re: others)	Mean: 1.87	Mean: 8.14
	SD: 2.88	SD: 10.81

In-degree (number of connections reported re: respondent by others)	Mean: 1.87	Mean: 8.14
	SD: 1.77	SD: 5.68

Trainee-supporter out-degree (number of connections reported by trainees regarding supporters)	Mean: 1.04	Mean: 8.26
	SD: 1.46	SD: 5.82

When we narrowed our focus to relationships between trainees and supporters, we found that at year 1, 94% of trainees reported informational exchange with supporters and 55% reported functional exchange with supporters. The average trainee-supporter out-degree at year 1 was 8.1 connections. In this network, the average number of outgoing connections with supporters was 2.3 for the lowest trainee-supporter out-degree tertile, 5.3 for the middle tertile, and 14.9 for the highest tertile. Trainee-supporter out-degree did not vary significantly between regions.

As seen in Table 5, trainee-supporter out-degree was positively associated with informational exchange, χ^2^(1, *N *= 23) = 74.93, p < 0.05. Those in the highest tertile of trainee-supporter out-degree also had the highest reports of informational exchange. We found a similar pattern for trainee-supporter out-degree and functional exchange, χ^2^(1, *N *= 23) = 81.31, p < 0.01.

**Table 5:** Reports of resource exchange by trainee-supporter connection level at year 1, contingency table analysis (n = 23)

	Informational: low exchange (%)	Informational: high exchange (%)	Functional: no exchange (%)	Functional: some exchange (%)
Trainee-supporter out-degree				

Low	20.00	6.67	19.51	9.76

Medium	13.33	13.33	14.63	14.63

High	20.00	26.67	19.51	21.95

				

Observed X^2^	74.93*		81.31**	

Key: ~ < 0.10, * p < 0.05, ** p < 0.01, *** p < 0.001

## Discussion and evaluation

We found substantial development of social networks within the context of a capacity-building program in healthcare management. Through involvement with the MHA program, participants developed professional connections with each other and with supporters, including faculty in Ethiopia and hospital executives in the United States of America. These connections supported valuable exchanges including information relating to hospital management and resources such as hands-on assistance.

The networks that developed through the first year of this program demonstrated several characteristics that have been shown to support resource exchange such as sufficient network density and connections between all or almost all members [26,32]. We found that the number of connections within the network was associated with likelihood of resource exchange, as hypothesized based on extant social network literature [10,40]. This level of growth and exchange may be expected in high-resource professional settings, such as corporations, academic institutions, or hospital systems in high-income countries [32,41] but is impressive in a low-resource setting given the level of investment required to support network development [40]. The growth is also notable given that network development was not an explicit goal of the training program.

Although the network growth and resource exchange are promising, limited resources for communication may have inhibited network development of some network members. We found that the network of program participants centered on a subset of individuals from the capitol city of Addis Ababa. The centralization of the network is important because the literature suggests that central members of a network have higher potential to access and utilize resources than their colleagues [10,42]. The pattern may reflect the relative ease with which individuals from Addis Ababa can interact, without communication impediments such as transportation and logistics that individuals from other regions may face. Information and communication technologies, such as mobile phones or internet, can mitigate challenges of physical distance and logistics in low-resource settings [25]. At the time of the study, reliable access to such technologies was limited for individuals working outside the Addis Ababa region [43], though these technologies may play an important role in network development in the future. Here, reduced opportunities to communicate and interact may have had a large impact on resource exchange in this network, as strong connections are required to support exchange of complex information [40].

We also saw evidence of the benefits of diverse connections for program participants and found that program participants were able to gain different categories of resources from different types of network members. This is likely a function of differential access to resources by individuals in different organizations and levels of power [10]. In a low-resource setting, other constraints may also be an important driver of resource exchange. For example, the material costs and logistical barriers associated with providing tangible support to colleagues may be too great for program participants. For mentors and educators, the costs of sharing both types of resources may be lower. The severe system-level constraints experienced by trainees were evident in a recent assessment of public hospitals engaged in a quality improvement initiative, including those represented by trainees in this program [44].

Experience with the MHA program suggests that programs to build human resource capacity in low-income countries can also increase network-based resources. However, given the common challenges of geography and limited communication technologies in such settings, social network development and resource exchange will likely be more effective if they are integrated as explicit goals of training programs to develop human resources for health. For instance, curricula can be developed to facilitate opportunities for developing new contacts. The focus on development of relationships should extend both to fellow trainees as well as supporters of the trainees, given the breadth of resources that can be accessed through diverse contacts. Another important lesson from the MHA experience is the importance of an enabling environment. This program was developed at the request of the Ethiopian government and was part of a broader effort to reform the healthcare system, such as adopting new hospital standards. This climate of organizational and system change was supportive of changing approaches to hospital management, and thus presented an environment in which social capital exchange was warranted and could have impact. Network development and social capital exchange may be particularly critical in low-resource settings as such networks can foster information and function exchanges in inexpensive ways.

There are several limitations that help place the results in context. First, although we had a high response rate, some trainees and supporters did not complete the survey potentially influencing our findings. However, we used out-degree as our independent variable, which is robust to missing data [45]. Second, the data are cross-sectional; thus causation cannot be assessed. However, a connection must exist between individuals before resources can be exchanged across that connection, so the directionality assumed seems plausible. Third, social desirability bias may have resulted in respondents over-reporting connections and/or resource exchanges, although we encouraged frank responses during survey administration. Despite these limitations, the study is a novel attempt to study network-based social capital in capacity-building programs targeting healthcare workforce development. Additionally, our assessment of resource exchange uses a broad view of social capital in public health settings, rather than the typical focus on communication patterns [46].

Developing human resources for health is an international priority in global health [47], and our paper highlights the importance of taking a broad view of outcomes of capacity-building programs. Capacity-building programs provide a unique opportunity to direct interactions between participants and potentially useful contacts through coursework, mentoring relationships, and other course-related activities. Active promotion of relationship-building by organizations and/or program developers can support diversity of contacts and development of strong channels for knowledge transfer [48-50]. In this way, the workforce and system will be better equipped to solve problems in healthcare by more effectively managing, accessing, and utilizing resources, thus truly building capacity [10,11].

## Conclusions

This analysis suggests that network-based social capital may be a useful addition to the goals and evaluation of capacity-building programs. As discussed by Hawe and colleagues [11], social capital deserves further attention in capacity-building efforts as it leaves the system under intervention with greater ability to tackle current issues as well as those outside the scope of the program and future issues. Through active development of diverse professional networks and investment in relationship-building within the context of system resource constraints, capacity-building programs can build stronger healthcare workforces in low- and middle-income countries.

## Competing interests

The authors declare that they have no competing interests.

## Authors' contributions

All authors were involved in study and survey instrument design. SR conducted the data analysis and drafted the manuscript. EHB, SK, and JM provided intellectual content and manuscript revisions. All authors read and approved the final manuscript.
